# Exploring the Asthma – Obesity Link Using Advanced Imaging Techniques

**DOI:** 10.33549/physiolres.935390

**Published:** 2025-02-01

**Authors:** Michaela KRIVOŠOVÁ, Romana BAROŠOVÁ, Eva LUKÁČOVÁ, Juliana HANUSRICHTEROVÁ, Nikolett NEMCOVÁ, Maroš KOLOMAZNÍK, Juraj MOKRÝ, Daniela MOKRÁ

**Affiliations:** 1 Biomedical Center Martin, Jessenius Faculty of Medicine in Martin, Comenius University in Bratislava, Martin, Slovak Republic; 2 Department of Physiology, Jessenius Faculty of Medicine in Martin, Comenius University in Bratislava, Martin, Slovak Republic; 3 Department of Molecular Biology and Genomics, Jessenius Faculty of Medicine in Martin, Comenius University in Bratislava, Martin, Slovak Republic; 4 Department of Pharmacology, Jessenius Faculty of Medicine in Martin, Comenius University in Bratislava, Martin, Slovak Republic

**Keywords:** Asthma, Obesity, Magnetic resonance imaging, Dual-energy, X-ray absorptiometry, Bioimpedance analysis

## Abstract

The global rise in obesity has emerged as a significant health concern, amplifying susceptibility to various diseases, including asthma. Epidemiological evidence demonstrates a higher prevalence of asthma among obese individuals, with obesity exacerbating asthma severity and control. This review aims to explore the interplay between asthma and obesity assessed by objective imaging methods and discusses the consistency between anthropometric and imaging methods. A literature search was conducted with the main keywords “asthma”, “obesity”, and “imaging techniques” using databases such as PubMed, Web of Sciences, and Scopus for the relevant articles published up to January 2024. The consistency between Body Mass Index (BMI), Waist Circumference (WC), and results from imaging techniques is uncertain. Unlike anthropometric methods, imaging methods provide us with the exact location of adipose tissue as well as fat and lean mass distinction, which can be further correlated with different airway parameters and respiratory system functions and dysfunctions. Studies indicate that the relationship between lung functions and obesity is more complex in females. Abdominal visceral fat is supposed to be the major asthma predictor already in the pediatric population. The connection between obesity and asthma is already evident in children and adolescents. Imaging methods can measure visceral and subcutaneous fat mass and both contribute to the association between obesity and lung functions. These methods are more accurate and reproducible but require more time and expertise.

## Introduction

**Fig. 1:**
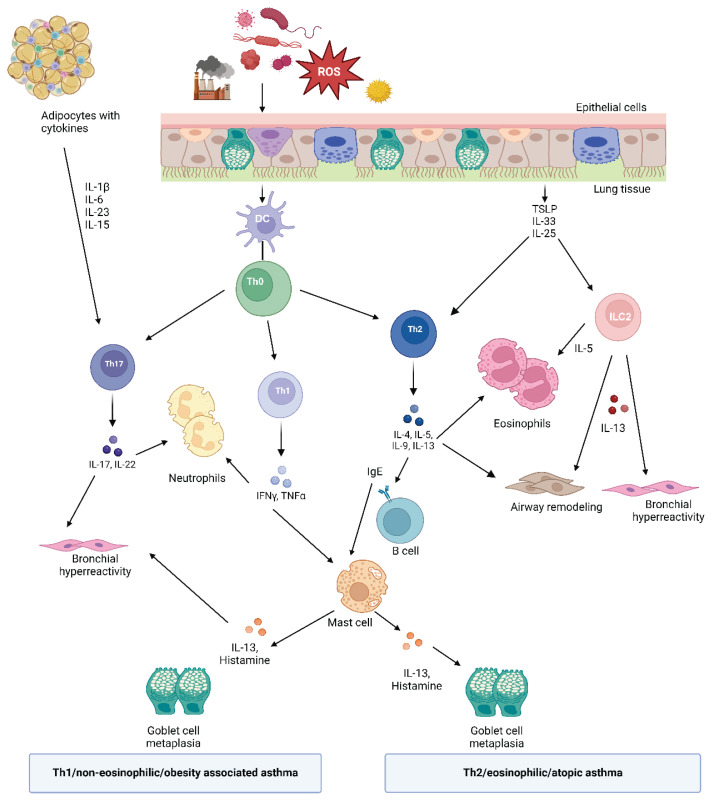
Scheme of the role of Th17, Th1, and Th2 cells in asthma. Once encounter allergens, pollutants, and respiratory viruses, airway epithelial cells produce a wide range of inflammatory cytokines. Moreover, excessive adipose tissue present in the body triggers a “low-grade inflammation”, with inflammatory cytokines contributing to airway inflammation. According to a subtype of asthma, various immune cells are activated and produce inflammatory cytokines and molecules causing typical asthma symptoms. Abbreviations: IFNγ: interferon gamma, IgE: immunoglobulin E, IL: interleukin, ROS: reactive oxygen species, TNFα: tumor necrosis factor alpha, TSLP: thymic stromal lymphopoietin.

The worldwide surge in obesity, which significantly increases susceptibility to various diseases including asthma [1], has become a global epidemic [2]. According to World Obesity Federation, obesity is considered to be chronic, relapsing, progressive disease that requires intervention [3]. Obesity alters respiratory functions in adults due to adipose mass compression and adipose tissue inflammation, contributing to respiratory dysfunction, irrespective of asthma presence [4,5]. Studies have shown that abdominal obesity increases intra-abdominal pressure, leading to restricted diaphragm movement and reduced lung volumes, such as forced expiratory volume (FEV) and forced vital capacity (FVC). In contrast, thoracic obesity, characterized by fat deposition around the chest, directly compresses the lungs and chest wall, further reducing lung compliance and ventilation efficiency. Epidemiological data show that asthma is more common in obese than lean individuals [6]; it more likely persists, requires more intensive treatment [4] and higher use of reliever medicines, and entails longer stays in hospital emergency room [7]. The risk of asthma is increased 1.4-fold in adults with BMI between 30.0 and 34.9 kg/m^2^ compared to those who have BMI<25 kg/m^2^ [8]. The association between obesity and asthma is so striking that ”obesity-associated asthma” represents a specific asthma phenotype, characterized by increased severity and poor asthma control [9] (Fig. 1). It may be further divided to early-onset atopic asthma (EOA), where asthma control does not improve significantly after weight loss, and late-onset non-atopic asthma (LONA), where the disease typically resolves with weight loss.

**Fig. 2:**
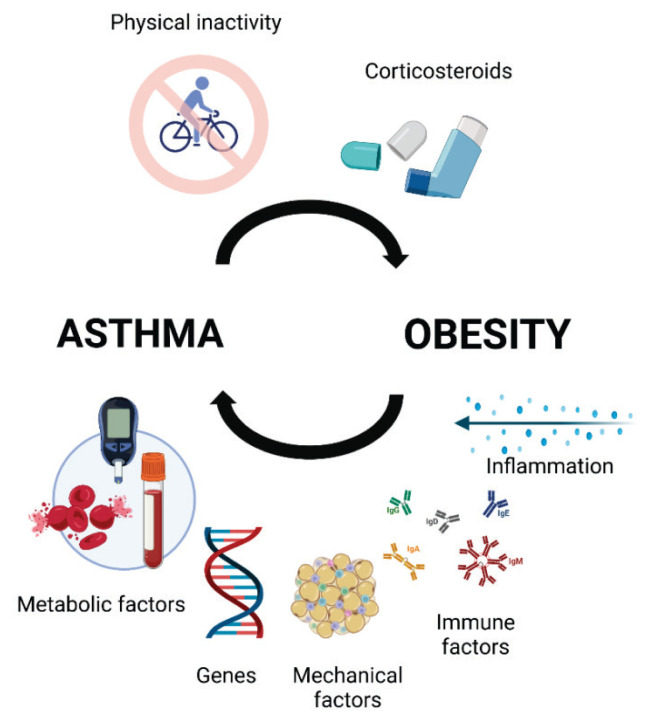
The vicious cycle of asthma and obesity. Obesity contributes to asthma worsening by several factors including mechanical, metabolic, inflammatory, immune, etc. On the other hand, asthmatics are more likely to develop obesity because of corticosteroid therapy and physical activity limitations.

The complex relationship between obesity and asthma can be understood through various interacting mechanisms, including genetic factors, diet and gut microbiome, presence of systemic inflammation, vitamin D deficiency, metabolic alterations, and changes in the lung anatomy and functions [6,10–12] (Fig. 2). Mechanically, adiposity represents extra load on thoracic cage leading to increased intra-abdominal pressure and hampered movements of the diaphragm [13]. Fat tissue in the thoracic region reduces chest cavity volume and diminishes chest wall movement. Obesity may affect the severity and nature of asthma *via* alteration in a degree of systemic inflammation. Adipose tissue is a metabolically active organ. Adipokines, molecules secreted by adipocytes, exert a pleiotropic effect. In the lean state, adipose tissue produces low concentrations of pro-inflammatory adipokines leptin and resistin, and cytokines such as interleukin (IL)-6, IL-8, and tumor necrosis factor (TNF)α. Simultaneously, high amount of anti-inflammatory adipokine adiponectin is produced. On the other hand, in the obese state, adipose tissue becomes hypertrophic and infiltrated with activated pro-inflammatory macrophages, which results in increased levels of pro-inflammatory cytokines and decreased adiponectin [14,15]. The count of macrophages in the adipose tissue of humans is usually low (4 %) but can reach up to 12 % in obesity [16].

Receptors for adiponectin and leptin are expressed on the airway epithelial cells, suggesting that direct effects of adipokines on the airway may be important in the pathogenesis of obesity-associated asthma. A positive correlation between fat leptin level and airway hyperreactivity has been reported [17]. According to a recent meta-analysis of 13 studies, low level of serum adiponectin is associated with higher asthma incidence. However, such result was found among adults, not children. On the other hand, higher leptin concentration was associated with asthma in both adults and children [18].

Visceral adipose tissue (VAT) is a smaller storage compartment for lipids compared to subcutaneous adipose tissue, with omental and mesenteric fat mechanistically linked to many of the metabolic disturbances and adverse outcomes associated with obesity. Hydrolysis of triglycerides within adipocytes releases free fatty acids, which are then transported in plasma to the sites where they can be utilized. Plasma levels of free fatty acids are often high in obese patients, reflecting enlarged adipose tissue mass [19]. A metabolomic study by Liu *et al*. showed that metabolomic profiles of obese asthma patients differentiated from profiles of lean asthma patients. The authors identified 28 significantly altered metabolites highlighting specific pathways, such as lipid metabolism, amino acid metabolism, and oxidative stress [20]. These findings suggest potential targets for personalized asthma treatment in obese patients.

Visceral adiposity significantly correlates with systemic levels of oxidative stress biomarkers [21,22]. Leptin has been shown to passively diffuse from blood to the airways and activate peripheral mononuclear cells, which promote oxidative burst and inflammatory response. An experimental study in murine alveolar macrophages shows that leptin elevated the production of arachidonic acid, prostaglandin E2 and leukotrienes in a dose-dependent manner [23]. Moreover, such metabolic inflammation seems to be a major factor contributing to complications of obesity [24].

The role of sex in obesity-associated asthma has also been discussed as different body composition and fat distribution between males and females may account for some sex differences in lung mechanics and asthma pathomechanisms [25–26]. For instance, Chen *et al*. showed that obesity is linked with increased risk of asthma and higher asthma severity in women but not in men [27]. These results were later confirmed by other studies, including a cluster analysis, which showed female predominance in the less atopic but corticosteroid-unresponsive asthma associated with obesity [28]. According to Sood *et al*., obesity and adipokines are more strongly associated with asthma in women than in men. The authors suggested that lean mass predicts asthma better than fat mass among females and possibly worse among men, both with and without adjustment for self-reported physical activity [26,29]. Recently, two obesity-associated phenotypes have been suggested: (1) obese asthmatics with an age of onset <12 years, poor control, atopic features with increased type 2 biomarkers, and significant airway hyperreactivity, who do not demonstrate sex predominance; and (2) obese asthmatics with an age of onset >12 years, fewer atopic features, lower airway hyperreactivity, better control, and normal level of type 2 biomarkers, who do show female predominance [30–32]. However, possible mechanisms of female predominance remain unclear and need further investigation, e.g., using metabolomics [33–34].

The aim of this article was to explore and analyse the association between asthma and obesity using objective imaging technique measurements as evidenced in existing studies.

## Materials and Methods

**Fig. 3:**
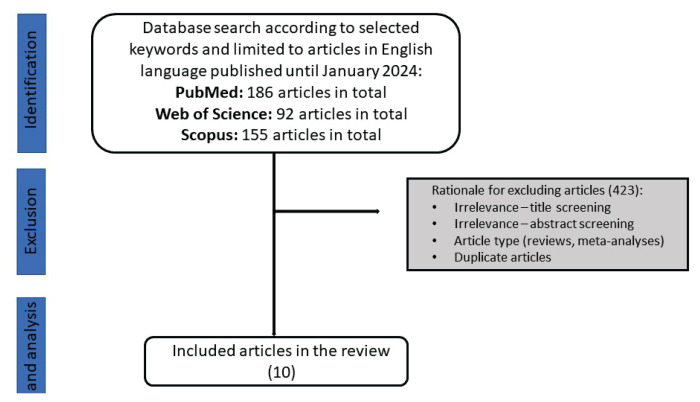
Scheme of the literature selection process.

A literature review was conducted to explore asthma-obesity link in the studies using imaging techniques. The search for relevant papers was done using three databases (PubMed, Web of Science, Scopus) up to January 2024. The following keywords were applied: “obesity” and “asthma”, and “imaging techniques”, “measurement”, “ultrasound”, “computed tomography (CT)”, “magnetic resonance imaging (MRI)”, “dual-energy X-ray absorptiometry, (DXA)”, “bioimpedance”. Only papers written in English language were included. The authors selected the most relevant papers according to the purpose of the study. The selection process is displayed in Figure 3, Table 1 summarizes the included articles.

## Measurement methods of obesity

To assess obesity, various measures can be employed. Majority of studies determining the association between asthma and obesity focused on the basic indicators such as BMI, calculated by dividing weight in kilograms by the square of height in meters. Subjects with a BMI≥30 kg/m^2^ are considered obese and those with a BMI 25–29.9 kg/m^2^ are considered overweight [35]. However, BMI is a composite measure of both lean and fat mass and it is frequently used as surrogate for adiposity mainly because of its feasibility [36]. It fails to accurately represent the distribution of body fat and often leads to misclassification, particularly in the individuals with intermediate BMI values [37]. Alternative anthropometric measures are considered, such as skin fold measurements, WC or Waist-to-Height Ratio (WHtR). A French cross-sectional population-based study revealed that abdominal obesity measured as WC was the strongest predictor of lung function impairment. It was positively related to both obstructive and restrictive ventilatory patterns, regardless of BMI [38]. In a HUNT study, the authors investigated correlation between BMI, WC, and asthma. Interestingly, general obesity was associated with asthma in both men and women; however, after additional adjustment for BMI, abdominal obesity remained a risk factor for asthma among females. The authors suggested that using both BMI and WC measures provides more valuable results compared to using only one of these parameters [39]. In a recent meta-analysis, anthropological measures WC, WHtR, Waist-to-Hip Ratio (WHR), and conicity index showed a positive association between abdominal obesity and asthma, similar in males and females [40].

Comparable to BMI, anthropometric measurements face limitations in distinguishing between lean tissue and body fat. They also lack the ability to discern variations between subcutaneous and visceral fat compartments. Therefore, using direct measures and the distribution of adiposity might be helpful in resolving the inconsistencies in the interplay between asthma/airway hyperresponsiveness and obesity. Notably, ultrasound, CT, MRI, and DXA emerge as advanced techniques for assessing abdominal adiposity. They are more accurate and reproducible than body fat distribution assessment by anthropometry. Measuring of both subcutaneous adipose tissue (SAT) and VAT is valuable as it determines the accumulation of abdominal fat [41]. Compared to SAT, VAT is believed to be more important in inflammation, metabolic derangement, insulin resistance, and dyslipidaemia. Both SAT and VAT should be established, since SAT and VAT differ in composition and function, and both contribute to the association between abdominal obesity and lung function [42]. The limitations of these methods are increased demands in terms of time, expertise, and costs, which could potentially hinder their widespread application in large-scale studies. Apart from these, each method has its specificities including disadvantages. For instance, DXA effectively detects larger fat deposits as fat mass; however, it inaccurately categorizes some smaller, highly metabolically active ectopic fat within skeletal muscle and internal organs as lean mass [26]. Using CT method has an advantage of better resolution but on the other hand, it provides greater ionizing radiation exposure. MRI technique needs extended imaging duration and specialized equipment for acquisition and analysis [43].

In the following sections we discuss obesity measurement by imaging methods and its relation to the lung functions in both adult and paediatric asthma patients.

## Obesity impact in asthma patients

In the cross-sectional study conducted by Sood *et al*. [44], fat mass index (FMI) and lean mass index (LMI) were measured by DXA approach and analysed together with BMI in 2000 participants in the 20-year follow up of the CARDIA study. Asthma was associated with elevated plasma level of F2-isoprostane, an oxidative stress marker; however, when adjusted for either gender or BMI, the association was not significant. Furthermore, BMI-asthma association was only seen among females, and this association was not determined by plasma F2-isoprostane concentration. The parameters FMI and LMI were likewise positively correlated with asthma in females. The study concluded that lean mass may be more important predictor compared to fat mass for asthma among females. It can be explained by the fact that the smaller ectopic fat deposits (lean mass) may be more relevant from inflammatory standpoint than the larger-physiological fat accumulation. It is contrary to the globally accepted belief that excess physiological fat (ectopic) drives the obesity among female asthmatics. Systemic oxidative stress was not proved to be associated with asthma and thus it failed to explain obesity-asthma link in this study [44].

Study conducted by Scott *et al*. [45] investigated a relationship between body composition, inflammation, and lung function in overweight and obese asthma patients, in which 44 participants with BMI 28–40 kg/m^2^ were enrolled. Lung functions were assessed and DXA scanning was performed. Inflammatory markers were analysed from induced sputum and venous blood samples. The study revealed that among females, android and thoracic fat tissue and total body lean tissue were inversely correlated with expiratory reserve volume (ERV), noting that for every 100 g decrease in android fat mass, there was a corresponding increase in ERV of 20 ml. On the other hand, in males, fat tissue did not correlate with lung function; however, there was a positive association between android and thoracic lean tissue and ERV. Lower body (leg and gynoid) lean tissue was positively associated with sputum neutrophil count in females, while leptin level was positively associated with android and thoracic fat tissue in males. Interestingly, while lean mass within the android and thoracic regions was associated with improved lung function in males, it had a negative or neutral effect in females, which confirms previous research by Sood *et al*. [44] and by Sutherland *et al*. [46] performed on healthy people. Therefore, although lean tissue itself is not proinflammatory, the presence of elevated intramyo-cellular lipid within lean tissue may exert proinflammatory effects since leptin is secreted 2–3-fold higher from subcutaneous compared to visceral adipose tissue. Moreover, the association between airway neutrophils and lower body lean tissue was observed. In conclusion, the study by Scott *et al*. indicates that reduction of central fat mass in females may lead to static lung function improvement [45].

Another method to stratify visceral and subcutaneous fat is ultrasound [47]. Study of Capelo *et al*. [48] collected data from 83 adult asthmatic women, 37 of whom had uncontrolled asthma. In this cross-sectional study the authors studied associations among abdominal adiposity distribution, asthma control, lung function, and cytokines. Asthma control was assessed according to GINA criteria; SAT and VAT were measured by ultrasound technique. Serum levels of adiponectin, IL-6, IL-8, transforming growth factor beta (TGF)-β, TNFα, and plasminogen activator inhibitor (PAI) levels were analysed. Peak expiratory flow (PEF) expressed as the percentage of the predicted value based on age, sex and height served as a parameter of asthma control and severity level. Anthropometric measures including WC, WHR and WHtR were assessed and calculated, respectively. After considering independent variables, including BMI, the findings indicate that asthma control impairment was linked to VAT and the VAT/SAT ratio, rather than anthropometric measures of central obesity. In an adjusted model, BMI and SAT were inversely associated with the adiponectin serum level, and VAT was associated with the IL-6 level. In conclusion, visceral obesity was negatively associated with asthma control and lung function, and positively associated with increased levels of IL-6 in women. The authors propose that future studies should consider women as a distinct group, including a control group, to determine whether visceral adipose inflammatory markers directly impact the uncontrolled asthmatic group. Moreover, compared with abdominal SAT, VAT is considered to be more metabolically active and can produce various hormones and cytokines including TNFα and IL-6 [48].

Yang *et al*. [49] investigated the association of abdominal fat accumulation with airway parameters measured *via* CT. In this study, 50 asthmatics (aged ≥40 years) were enrolled. Airway parameters including lumen diameter, lumen area, wall area (WA), bronchial wall thickness, total area (TA) and WA/TA percentage were assessed. As obesity measures, visceral fat area (VFA), subcutaneous fat area (SFA), and total fat area (TFA) were obtained. Elevated visceral to subcutaneous fat area ratio (VFA/SFA ≥0.4) was estimated to express excess of visceral fat efficiently. In this study, abdominal fat was associated with asthma based on fat distribution, with visceral fat potentially contributing to bronchial luminal narrowing. On the other hand, subcutaneous fat may be related to thickening of bronchial wall. Thus, the results suggest that different locations of adipose tissues within the body of asthmatics might be related to different airway parameters. Interestingly, in this study only about half of the subjects had BMI 25 kg/m^2^ or higher, and it seemed that even non-obese subjects were affected by their fat location [49].

The Japanese study of Goudarzi *et al*. [41] aimed to clarify the differential impact of several obesity-related indices, including BMI, WC, and abdominal visceral and subcutaneous fat, on asthma symptoms. Anthropometric measures were evaluated, and abdominal CT scans performed in 206 asthmatics. The authors also assessed quality of life (QOL) score and identified well-known obesity-associated comorbidities, such as gastroeso-phageal reflux disease (GERD), depression, and daytime somnolence, in order to evaluate the potential impact of obesity on asthma symptoms. The study shows that all four measured obesity-related indices were significantly associated with QOL in females, while only the abdominal visceral fat mass measured by CT was significantly associated with QOL regardless of sex. However, only the visceral fat area was statistically inversely associated with QOL in males. Only abdominal visceral fat showed association with higher GERD and depression scores. Although all obesity indices showed inverse association with functional residual capacity, only visceral fat area had a significant inverse association with FEV1 predicted, independent of other obesity measures. These results indicate that visceral adiposity holds greater clinical relevance for asthma outcomes compared to BMI or WC. However, one of the limitations of the study was a small proportion of obese participants [41].

Another method for body fat percentage (BFP) measurement is bioimpedance. It reaches lower accuracy than DXA technique; however, equivalent results were shown [43]. Work of Deng *et al*. [50] investigated the effect of VFA on the clinical features of asthma as well as future risk of exacerbation in 12-month prospective study. The measurement of VFA, fat mass in kg (FM) and BFP were done using bioimpedance. BMI and WHR were also calculated. Total and differential sputum cell counts and sputum inflammatory biomarkers (IL-1β, IL-4, IL-6, IL-8, IL-17A, TNFα) and IgE levels were analysed. The enrolled patients were divided into the low VFA and high VFA groups based on VFA median levels at baseline. In this study, the authors found out that the level of VFA was associated with specific clinical and inflammatory characteristics of asthma. Furthermore, VFA, as an independent risk factor, was associated with an increased risk of exacerbations. The results of the study also suggest that VFA was significantly associated with age in the female rather than the male patients and that BMI and WHR cannot accurately reflect VFA [50].

## Asthma-obesity link in pediatric population

The obesity-asthma link in pediatric population is driven mainly by visceral fat, independent of total fat mass [51]. DXA technique may be used to measure general fat while MRI is utilized to measure organ fat including subcutaneous fat index, visceral fat index, pericardial fat index, and liver fat fraction. Higher visceral fat index, independent of fat mass index, was associated with higher risk of asthma and no other organ fat measures were independently associated with lung function or asthma. Also, in young healthy participants the lung function was found to be highly associated with abdominal fat distribution. Vital capacity index (VCI) was more negatively correlated with VFA for men in comparison with SAT for women, respectively, in comparison with other tested indices [43].

Another study that included both asthmatic and non-asthmatic obese adolescent participants identified a significant association between food consumption and lung function improvement in both groups. After one-year follow-up of nutritional therapy there was a reduction in BMI, body fat percentage, visceral and subcutaneous fat and an increase in lean mass as well as in all lung function variables in both groups, except the FEV1/FVC ratio in non-asthmatic patients [52].

## Conclusions

In conclusion, most of aforementioned studies indicate that relationship between respiratory function, inflammation, and obesity is more complex in females compared to males. Frequently, fat and lean mass are associated only with asthmatic female patients. However, some studies found association between fat mass and asthma in females and a positive association between lean mass and lung functions in males. Not all the studies confirmed a consistency between anthropometric and imaging techniques of obesity measurement in association with asthma. Fairly equivalent results were shown using BIA and DXA; however, more studies should confirm its reproducibility. Different locations of fat mass can be related to different airway parameters and both subcutaneous and visceral fat can provide an important information. Abdominal visceral fat is the major asthma predictor of obesity indices and furthermore, it showed a significant association with decreased quality of life in asthma patients. The connection between obesity and asthma is equally evident in the pediatric population, with visceral fat being a primary factor to consider. Nutrition therapy can improve both the obesity indices and lung functions.

## Figures and Tables

**Table 1:** Summary of included articles.

Authors	Year	Article	Obesity measurement method
Sood *et al*.	2009	Obesity-Asthma Association	DXA
Scott *et al*.	2012	Relationship between body composition, inflammation and lung function in overweight and obese asthma	DXA
Bubnov *et al*.	2019	Asthma association with metabolic syndrome and obesity: relevance of ultrasound evaluation of visceral fat and posture	Ultrasound
Capelo *et al*.	2016	Visceral adiposity is associated with cytokines and decrease in lung function in women with persistent asthma	Ultrasound
Yang *et al*.	2018	Association Between Airway Parameters and Abdominal Fat Measured *via* Computed Tomography in Asthmatic Patients	CT
Goudarzi *et al*.	2019	Impact of Abdominal Visceral Adiposity on Adult Asthma Symptoms	CT
Huang *et al*.	2019	Effects of fat distribution on lung function in young adults	Bioimpedance
Deng *et al*.	2020	Visceral obesity is associated with clinical and inflammatory features of asthma: A prospective cohort study	Bioimpedance
Mensink-Bout *et al*.	2020	General and Organ Fat Assessed by Magnetic Resonance Imaging and Respiratory Outcomes in Childhood	DXA, MRI
Rodrigues *et al*.	2019	Nutrient intake is a predictor of lung function in obese asthmatic adolescents undergoing interdisciplinary therapy	Ultrasound

Abbreviations: CT: computed tomography, DXA: dual-energy X-ray absorptiometry, MRI: magnetic resonance imaging.
